# Impediments and Facilitators to Physical Activity and Perceptions of Sedentary Behavior Among Urban Community Residents: The Fair Park Study

**DOI:** 10.5888/pcd10.130125

**Published:** 2013-10-31

**Authors:** Kerem Shuval, Emily T. Hébert, Zoveen Siddiqi, Tammy Leonard, Simon Craddock Lee, Jasmin A. Tiro, Katharine McCallister, Celette Sugg Skinner

**Affiliations:** Author Affiliations: Emily T. Hébert, Zoveen Siddiqi, University of Texas School of Public Health, Dallas and Austin, Texas; Tammy Leonard, University of Texas at Dallas, Dallas, Texas; Simon Craddock Lee, Jasmin A. Tiro, Katharine McCallister, Celette Sugg Skinner, University of Texas Southwestern Medical Center and the Simmons Cancer Center, Dallas, Texas. Dr Shuval is also affiliated with the University of Texas School of Public Health and the Simmons Cancer Center at the University of Texas Southwestern Medical Center, Dallas, Texas.

## Abstract

**Introduction:**

Insufficient physical activity is an established risk factor for numerous chronic diseases and for premature death. Accumulating evidence reveals that prolonged sedentary time is detrimental, independent of the protective effects of physical activity. Although studies have explored correlates of physical activity among ethnic minority populations, few have examined factors related to sedentary behavior. Therefore, we conducted a preliminary investigation into urban adults’ perceptions of sedentary behavior alongside perceived barriers and enablers to physical activity.

**Methods:**

In-depth semi-structured interviews were used to evaluate perceptions of physical activity and sedentary behavior in a sample of low-income, ethnic minority adults. The framework approach guided researchers in analyzing the qualitative data.

**Results:**

Participants were well aware of the positive health benefits of physical activity. However, most admitted not regularly engaging in physical activity and cited numerous barriers to activity, such as lack of time, insufficient finances, and neighborhood crime. Enablers included weight loss, the presence of social support, and the availability of safe parks conducive to exercise. In comparison, participants were primarily unfamiliar with the term “sedentary behavior” and did not perceive a relationship between sedentary behavior and health outcomes.

**Conclusion:**

Our findings illustrate the need to increase the awareness of negative health implications of prolonged sedentary time while continuing to address the multiple impediments to physical activity as a way to combat chronic disease.

## Introduction

Insufficient physical activity is a risk factor for death related to all causes, cardiovascular disease, and cancer; it is also a risk factor for type 2 diabetes, hypertension, dyslipidemia, obesity, myocardial infarction, stroke, osteoporosis, osteoarthritis, breast cancer, colon cancer, and depression (1). Although the health benefits of physical activity are well known, many Americans do not meet the public health guidelines, that is, engage in 150 minutes or more a week of moderate-intensity aerobic physical activity or 75 minutes or more of vigorous-intensity activity (or an equivalent combination).

On the basis of data from the National Health Interview Survey, less than half of those surveyed (48.2%) met aerobic physical activity guidelines in early 2012 (2). When examining these data by race/ethnicity, non-Hispanic blacks or African Americans (African Americans) and Hispanics were less likely to meet public health recommendations than were non-Hispanic whites (whites): 36.4%, 41.4%, and 52.2%, respectively (2). This disparity might stem from lack of culturally appropriate interventions or differences in physical activity self-efficacy and knowledge, income, education level, physical environment, neighborhood crime, access to exercise facilities, social support, or neighborhood social cohesion (3–5).

Concurrent with the decreased likelihood of meeting physical activity guidelines, the prevalence of some chronic conditions (eg, type 2 diabetes) is higher among African Americans and Hispanics than whites. For example, African Americans and Hispanics over the age of 20 years have higher prevalences of obesity than their white counterparts (49.5%, 39.1%, and 34.3%, respectively) on the basis of data from the National Health and Nutrition Examination Survey (6). Similarly, whites had lower rates of diagnosed diabetes than Hispanics and African Americans (7.1%, 11.8%, and 12.6%, respectively) (7). These examples emphasize the need to promote physical activity among ethnic minority populations to delay or prevent the onset of chronic disease.

In addition to insufficient physical activity as an established risk factor for disease, accumulating evidence indicates that prolonged sedentary time increases the risk of illness and death from chronic disease independent of the protective effects of physical activity (8–10). Sedentary behavior, that is, activities in a sitting or reclining posture requiring low levels of energy expenditure (11), has been linked to higher odds of obesity and type 2 diabetes and to death from all causes and deaths from cardiovascular disease (12,13). Hence, sedentary behavior should be a specific focus of investigation (14). We assess urban adults’ perceptions of sedentary behavior alongside physical activity. The primary aims of the study are twofold: 1) to explore impediments and enablers to physical activity among study participants, and 2) to conduct a preliminary investigation into attitudes toward sedentary behavior and their perceived association with health.

## Methods

### Design and participants

We conducted a qualitative study, well-suited for assessing perceptions and behaviors of participants in their natural environment (15,16), to examine factors affecting physical activity and perceptions of sedentary behavior among adult participants in the Fair Park Study. This study, described in detail elsewhere (17,18), aimed to assess the effect of public investment on socioeconomic and health factors of residents in a low-income community in South Dallas, Texas. Before study initiation, ethical approval was obtained from the institutional review boards of the University of Texas Southwestern Medical Center and the University of Texas Health Science Center at Houston. Data for the investigation are from the second wave of the Fair Park Study, which included information on participants’ lifestyle behaviors (eg, physical activity) obtained via questionnaire (n = 496).

Of those completing the survey, we approached a convenience sample of 91 individuals and asked them to participate in a pedometer study; of these, 11 were not interested, 3 were not proficient in English, 1 had a transportation problem, and 1 experienced technical difficulties with the pedometer. This resulted in 75 participants who expressed an interest, enrolled in the study, and received a pedometer to determine daily step counts. Sixty-four of those provided with a pedometer returned it to the research team. Researchers approached participants on return of the pedometers to gauge their interest in taking part in qualitative interviews. The research team attempted to purposively sample an equal representation of both men and women to identify potential differences in opinion by sex. A total of 28 were approached; of these, 2 declined to participate and 1 agreed but did not show up for the interview.

The final 25 participants were primarily African American (82%) who, on average, were aged 42 years (range, 30–54 years). Nearly two-thirds (68%) were high school graduates, slightly more than half (52%) were women, 60% had children under 18 years living in the household, 40% were employed, and the annual household income of most (72%) was below $30,000. Additionally, 68% were overweight or obese, and their average daily step count was 4,096 steps (standard deviation, 2,703), as measured by pedometers. The qualitative study sample was not statistically different from the full sample (n = 496) with respect to sex, income, employment, marital status, education, or body mass index.

### Qualitative interviews, procedures, and analysis

The research team explained the study aim before the interviews and provided potential interviewees with an information sheet describing the study protocol; verbal consent was obtained from those agreeing to participate, and participants received a $15 gift card at the completion of the interview. Semi-structured interviews, conducted during October and November 2009, lasted approximately an hour and were held in a designated room at the University of Texas at Dallas Field Research Center in Fair Park. We sought information on interviewees’ perceptions of physical activity within the socioecological framework (19) and an initial insight into perceptions of sedentary behavior and health. Specifically, interviewees were asked to describe their attitudes toward physical activity; to indicate if they felt they were sufficiently active and if physical activity is linked to health; to describe attitudes toward the pedometers used and whether they affected their physical activity; and to describe barriers and enablers to physical activity on the individual (eg, lack of time), social (eg, social support), and environmental (eg, neighborhood characteristics, exercise facilities) levels. In addition, participants were asked whether they were familiar with the term “sedentary behavior” (if not, it was explained to them) and asked to describe their lifestyle in this context; whether they believed there is a relation between prolonged sedentary time and health; and which factors affect sedentary time.

All interviews with participants were audio-recorded and transcribed verbatim. Two researchers (K.S., Z.S.) scrutinized the data independently to enhance trustworthiness (20). Consensus between researchers was high (80% on themes) and any disagreement was resolved through peer debriefing until reaching 100% consensus. The framework approach guided the data analysis process: researchers first read transcripts several times to immerse themselves in the data, and then created thematic charts based on a priori and new emerging themes (21). The final stage of analysis consisted of interpreting the data while taking into account the study objectives.

## Results

The following 4 main themes emerged during the analysis: perceptions of sedentary behavior, perceptions of physical activity, barriers to physical activity, and facilitators to physical activity. Because many themes were shared by both men and women and all age groups, results were not stratified by these variables.

### Perceptions of sedentary behavior

Although the term “sedentary behavior” was unfamiliar to most, 1 participant defined sedentary behavior as “stagnated” (male, participant number 1; ie, no. 1) while another defined it as “sit around and don’t do nothing” (female, no. 2). Once the term “sedentary behavior” was defined as excessive sitting to those who were unfamiliar with this terminology, all understood and most reported being sedentary. Participants reported either sleeping or watching TV: “Like today, other than having this appointment I’ll be sitting at the house watching TV” (male, no. 3); “I sleep . . . I mean, sleep and watch TV” (female, no. 4); “I’m not always watching TV, I might be sitting on the couch . . . reading” (male, no. 5); “(I’m) sitting on my behind all day. Ride all day . . . and that contributes to me being lazy . . . the truck wears me out. So once I get out of the truck after a 10-hour day, 12 hours maybe, I’m pretty much worn” (male, no. 6). A few, however, noted that they were constantly “moving about” or attempting not to be sedentary: “I stay moving, you know, there is really no time to sit down for me, my kids keep me moving” (female, no. 7); or “I try to visit people and stuff to keep from just sitting and looking at the TV all day” (female, no. 8).

### Perceptions of physical activity

Although participants did not link increased sitting to adverse health outcomes, they did underscore the role habitual physical activity plays in achieving both physical and mental health. Physical activity was perceived as a way to improve longevity: “It (physical activity) means to . . . live longer” (male, no. 9); to achieve cardiovascular health: “Physical activity is good for my heart and my blood circulation” (male, no. 10); to facilitate weight loss: “I have to lose weight so I think it’s good for me exercising” (female, no. 11); and to bestow a sense of well-being and mental health: “I feel better when I am active” (female, no. 12) , “It (physical activity) affects you mentally” (female, no. 13), and “I could stand to exercise more . . . I think I’d be less stressed” (male, no. 14). Despite being cognizant of the health benefits of physical activity, almost all reported no leisure-time physical activity; however, a few noted work-related or household activity: “I don’t exercise the way I should. . . . I always say I get enough exercise at work” (male, no. 14); and “I don't do no exercise . . . because I get my exercise from moving the furniture around in the house” (female, no. 8). Some noted that they are contemplating becoming more active to achieve health benefits: “I need to be more active, I know that because I’m going through some medical issues, and I do need to lose more than just a couple of pounds. But no I am not physically fit . . . not as much as I need to be” (female, no. 15). Some reported that wearing pedometers made them more aware of the amount of physical activity they were engaged in and prompted them to either increase their activity or contemplate doing so. For example, a participant said, “When I wore it (pedometer) . . . it made me want to go faster . . . do more exercise, do more walking . . . make the numbers go up” (female, no. 16). Another participant said, “I need to be more physically active and healthier than I am, but I'm not. But the week that I wore the thing (pedometer), it motivated me. It really did” (female, no. 15).

### Barriers to physical activity

Participants described barriers to physical activity on both individual and socioenvironmental levels. On an individual level, fatigue, lack of motivation, and time were cited as impediments. One participant indicated, “I just don't have the energy” (female, no. 4), and another mentioned, “I never find the time to do it (physical activity), that’s my problem” (male, no. 6). Participants also referred to physical disabilities and other health ailments as barriers to activity, such as coronary heart disease and musculoskeletal injuries: “I am retired and my back is bad so I don’t do a lot of exercise” (male, no. 3); “I can’t walk because my legs be hurting” (female, no. 17); and “the heart situation . . . the chest pain” (female, no. 15).

In addition, participants also elaborated on socioenvironmental factors impeding their daily activity, such as lack of exercise facilities and parks and neighborhood crime. For example, participants observed, “We ain’t really got no gym over here that we can actually go to and work out” (male, no. 9) and “My kids don't even go to the parks like we used to when we were growing up because there’s nothing really there” (female, no. 18). Moreover, participants perceived their neighborhood as unsafe because of crime: “South Dallas, it’s not really safe; if I felt more safe I would do more activities” (female, no. 16); and “It’s a high crime area. . . . You can’t just get out there and walk in your neighborhood” (male, no. 19). In addition, many participants cited low income resulting from unemployment or underemployment as a substantial impediment: “In order to do that (exercise) you either [need] to be able to afford the equipment or be able to afford being able to go somewheres to doing it” (male, no. 9), and: “People just trying to go to work and . . . pay bills. . . . With higher education comes higher wages. With high wages may come the desire to have a better quality of life and once you desire to have a better quality of life then I think exercise will come” (male, no. 14).

### Facilitators of physical activity

A primary individual facilitator to physical activity cited was enhanced physique and weight loss associated with an active lifestyle: “I want to be healthy and I want to lose weight, I want to live, I want to look good for my age” (female, no. 16); “I would like to do exercises that would tone my muscles and keep my body toned ’cause as you get older parts of your body, like arms getting flabby” (female, no. 2). In addition, on an interpersonal level, social support from family or friends was believed to be an important enabler: “I got to get a push. I get a call from my friend and she’s like, ‘Are you going?’ and I’m like, ‘Okay, I wasn’t going but I’m going now’” (female, no. 12); “[If] I had a workout partner and possibly a workout program . . . I would be . . . willing to participate in more physical activity” (female, no. 4). Additionally, having children at home was perceived as a facilitator to household physical activity: “I do a lot of physical activity because . . . I have kids so I stay active with them. I’m cooking, I’m washing clothes, washing dishes, cleaning” (female, no. 8). Furthermore, participants reported adhering to physicians’ advice to adopt a physically active lifestyle as a means to promote general health and to lose weight: “My doctor told me it was good for me to walk” (female, no. 17); “I’m overweight and according to the doctor I have to lose weight” (female, no. 11). Furthermore, at the community level, participants stressed the importance of the following enablers to physical activity: presence and easy access to parks and open spaces, availability of nearby recreational and exercise facilities, and the availability of structured exercise classes at minimum or no cost. For example, 1 participant noted, “There needs to be somewhere that people can go, there needs to be a fitness center or . . . recreation center . . . it needs to be offered and it needs to be free . . . make that available or affordable to people” (male, no. 14). Another participant said, “On that vacant lot put a park, put a few benches, some swings, a slide, something, you know, make it to where you know people can do something there besides stand around” (female, no. 12).

## Discussion

To our knowledge, this is the first study to assess adults’ perceptions of sedentary behavior in a low-income, ethnic minority population. Although multiple studies have explored attitudes pertaining to physical activity (22,23), few have specifically focused on sedentary behaviors despite the fact that prolonged sedentary behavior is independently associated with adverse effects on health (24). Our findings indicate that participants were not able to provide an accurate definition of sedentary behavior, nor did they see any relationship between this type of maladaptive behavior and health outcomes. In comparison, participants were well aware of the role that physical activity plays in chronic disease prevention, and many expressed an interest in becoming physically active despite not being able to regularly exercise because of a multitude of barriers. Hence, our findings underscore the need to increase awareness of the health risks associated with excessive sitting while promoting the adoption and maintenance of a physically active lifestyle by addressing barriers to activity among this population (1,8,9,25).

Our results pertaining to physical activity are consistent with the literature, with impediments to physical activity reported both at the individual and the socioenvironmental levels (22). On an individual level, fatigue, lack of motivation, and income were cited as impediments. Participants also felt that environmental factors such as lack of exercise facilities and prevalent neighborhood crime hindered their activity. Additionally, multiple enablers to physical activity were cited, such as a desire to be healthy, social support from family or friends, and environmental elements such as nearby recreational and exercise facilities. As in our study, Siddiqi et al in a systematic review found that lack of time and self-efficacy and unsafe neighborhoods were commonly cited barriers to activity among African American adults (22).

Our study, however, extends previous research by exploring perceptions of sedentary behavior among participants in a predominately African American sample living in an impoverished neighborhood, who are at increased risk for illness and death from chronic disease (26). This initial exploration did not lead to examination of factors affecting sedentary behavior, because physical inactivity and prolonged sitting were not perceived as independent and distinct risk factors for disease. It is plausible that some factors affecting participants’ physical activity (eg, neighborhood crime) also contributed to increased sedentary time. Exploration of correlates of sedentary behavior among this population is warranted. Mabry et al (27) similarly assessed perceptions of sedentary behavior; however, that study was among a smaller (n = 10) and select sample of public health professionals in Oman. Consistent with our study, participants in Mabry et al’s study also appeared to be unable to distinguish between insufficient physical activity and sedentary behavior, and few factors affecting sitting behavior were described.

Our study has several limitations. Because of insufficient research investigating perceptions and attitudes toward sedentary behavior, it is unclear whether this lack of awareness of sedentary behavior and its health consequences is reflective of the general population or is limited to our study sample. In addition, in comparison to quantitative studies, the study sample size used is small (n = 25), with limited generalizability; however, the sample is likely similar to other low-income, ethnic minority communities elsewhere in the United States.

The public health agenda for modifying sedentary behavior as a means to prevent chronic disease is regarded as a new pursuit, with few clinical and population-level guidelines available for adults (28); guidelines for children and adolescents are more prevalent (27,29). Thus, despite the numerous studies linking increased sitting time to higher risks for chronic conditions and premature death, additional research is warranted on how to translate this evidence into public health practice. Our findings stress the need to increase awareness of the adverse health effects of prolonged sitting before focusing on implementing interventions aimed at changing this behavior (30). Additional research is needed to more elaborately explore determinants of sedentary behavior, which will inform program planners in developing sedentary behavior interventions.

## References

[1] US Department of Health and Human Services. 2008 physical activity guidelines for Americans. October 2008. http://www.health.gov/paguidelines. Accessed June 16, 2013.

[2] Ward B , Schiller J , Freeman G , Peregoy J . Early release of selected estimates based on data from the January–September 2012 National Health Interview Survey. March 2013. http://www.cdc.gov/nchs/nhis.htm. Accessed June 16, 2013.

[3] Gordon-Larsen P , Nelson MC , Page P , Popkin BM . Inequality in the built environment underlies key health disparities in physical activity and obesity. Pediatrics 2006;117(2):417–24. 10.1542/peds.2005-0058 16452361

[4] Powell LM , Slater S , Chaloupka FJ . The relationship between community physical activity settings and race, ethnicity and socioeconomic status. Evidence-Based Preventive Medicine 2004;1(2):135–44.

[5] Ahmed NU , Smith GL , Flores AM , Pamies RJ , Mason H , Woods KF , Racial/ethnic disparity and predictors of leisure-time physical activity among US men. Ethn Dis 2005;15(1):40–52. 15720048

[6] Flegal KM , Carroll MD , Kit BK , Ogden CL . Prevalence of obesity and trends in the distribution of body mass index among US adults, 1999–2010. JAMA 2012;307(5):491–7. 10.1001/jama.2012.39 22253363

[7] National diabetes fact sheet: national estimates and general information on diabetes and prediabetes in the United States. Atlanta (GA): US Department of Health and Human Services, Centers for Disease Control and Prevention; 2011.

[8] Healy GN , Dunstan DW , Salmon J , Shaw JE , Zimmet PZ , Owen N . Television time and continuous metabolic risk in physically active adults. Med Sci Sports Exerc 2008;40(4):639–45. 10.1249/MSS.0b013e3181607421 18317383

[9] Dunstan DW , Barr EL , Healy GN , Salmon J , Shaw JE , Balkau B , Television viewing time and mortality: the Australian Diabetes, Obesity and Lifestyle Study (AusDiab). Circulation 2010;121(3):384–91. 10.1161/CIRCULATIONAHA.109.894824 20065160

[10] Hamilton MT , Healy GN , Dunstan DW , Zderic TW , Owen N . Too little exercise and too much sitting: inactivity physiology and the need for new recommendations on sedentary behavior. Curr Cardiovasc Risk Rep 2008;2(4):292–8. 10.1007/s12170-008-0054-8 22905272 PMC3419586

[11] Tremblay MS , Colley RC , Saunders TJ , Healy GN , Owen N . Physiological and health implications of a sedentary lifestyle. Appl Physiol Nutr Metab 2010;35(6):725–40. 10.1139/H10-079 21164543

[12] Katzmarzyk PT , Church TS , Craig CL , Bouchard C . Sitting time and mortality from all causes, cardiovascular disease, and cancer. Med Sci Sports Exerc 2009;41(5):998–1005. 10.1249/MSS.0b013e3181930355 19346988

[13] Ford ES , Caspersen CJ . Sedentary behaviour and cardiovascular disease: a review of prospective studies. Int J Epidemiol 2012;41(5):1338–53. 10.1093/ije/dys078 22634869 PMC4582407

[14] Katzmarzyk PT . Physical activity, sedentary behavior, and health: paradigm paralysis or paradigm shift? Diabetes 2010;59(11):2717–25. 10.2337/db10-0822 20980470 PMC2963526

[15] Kuper A , Reeves S , Levinson W . An introduction to reading and appraising qualitative research. BMJ 2008;337(7666):404–7. 18687727 10.1136/bmj.a288

[16] Shuval K , Harker K , Roudsari B , Groce NE , Mills B , Siddiqi Z , Is qualitative research second class science? A quantitative longitudinal examination of qualitative research in medical journals. PLoS ONE 2011;6(2):e16937. 10.1371/journal.pone.0016937 21383987 PMC3044713

[17] Leonard T , Shuval K , de Oliveira A , Skinner CS , Eckel C , Murdoch JC . Health behavior and behavioral economics: economic preferences and physical activity stages of change in a low-income African-American community. Am J Health Promot 2013;27(4):211–21. 10.4278/ajhp.110624-QUAN-264 23448410 PMC4225127

[18] Shuval K , Leonard T , Murdoch J , Caughy Mo , Kohl HW 3rd , Skinner CS . Sedentary behaviors and obesity in a low income, ethnic-minority population. J Phys Act Health 2013;10(1):132– 6. 22398752 PMC3597085

[19] Sallis JF , Owen N , Fisher EB . Ecological models of health behavior. In: Glanz K, Rimer BK, Viswanath K, editors. Health behavior and health education: theory, research, and practice. San Francisco (CA): John Wiley and Sons Inc; 2008.

[20] Mays N , Pope C . Rigour and qualitative research. BMJ 1995;311(6997):109–12. 10.1136/bmj.311.6997.109 7613363 PMC2550154

[21] Pope C , Ziebland S , Mays N . Qualitative research in health care. Analysing qualitative data. BMJ 2000;320(7227):114–6. 10.1136/bmj.320.7227.114 10625273 PMC1117368

[22] Siddiqi Z , Tiro JA , Shuval K . Understanding impediments and enablers to physical activity among African American adults: a systematic review of qualitative studies. Health Educ Res 2011;26(6):1010–24. 10.1093/her/cyr068 21873458

[23] Mathews AE , Laditka SB , Laditka JN , Wilcox S , Corwin SJ , Liu R , Older adults’ perceived physical activity enablers and barriers: a multicultural perspective. J Aging Phys Act 2010;18(2):119–40. 20440026 10.1123/japa.18.2.119

[24] Matthews CE , George SM , Moore SC , Bowles HR , Blair A , Park Y , Amount of time spent in sedentary behaviors and cause-specific mortality in US adults. Am J Clin Nutr 2012;95(2):437–45. 10.3945/ajcn.111.019620 22218159 PMC3260070

[25] Bauman AE , Reis RS , Sallis JF , Wells JC , Loos RJ , Martin BW , Correlates of physical activity: why are some people physically active and others not? Lancet 2012;380(9838):258–71. 10.1016/S0140-6736(12)60735-1 22818938

[26] Shuval K , DeVahl J , Tong L , Gimpel N , DeHaven MJ , Lee JJ . Anthropometric measures, presence of metabolic syndrome, and adherence to physical activity guidelines among African American church members, Dallas, Texas, 2008. Prev Chronic Dis 2011;8(1):A18. 21159230 PMC3044029

[27] Mabry RM , Al-Busaidi ZQ , Reeves MM , Owen N , Eakin EG . Addressing physical inactivity in Omani adults: perceptions of public health managers. Public Health Nutr 2013:1–8. 10.1017/S1368980012005678 23347388 PMC10282297

[28] Shuval K , DiPietro L , Skinner CS , Barlow CE , Morrow J , Goldsteen R , ‘Sedentary behaviour counselling’: the next step in lifestyle counselling in primary care; pilot findings from the Rapid Assessment Disuse Index (RADI) study. Br J Sports Med 2012. 10.1136/bjsports-2012-091357 22976910 PMC4229046

[29] Canadian physical activity guidelines and Canadian sedentary behaviour guidelines. http://www.csep.ca/guidelines. Accessed June 16, 2013.

[30] Owen N , Sugiyama T , Eakin EE , Gardiner PA , Tremblay MS , Sallis JF . Adults’ sedentary behavior determinants and interventions. Am J Prev Med 2011;41(2):189–96. 10.1016/j.amepre.2011.05.013 21767727

